# Cadmium down-regulates expression of XIAP at the post-transcriptional level in prostate cancer cells through an NF-κB-independent, proteasome-mediated mechanism

**DOI:** 10.1186/1476-4598-9-183

**Published:** 2010-07-09

**Authors:** Konstantin Golovine, Peter Makhov, Robert G Uzzo, Alexander Kutikov, David J Kaplan, Eric Fox, Vladimir M Kolenko

**Affiliations:** 1 Division of Urological Oncology, Department of Surgery, Fox Chase Cancer Center, Philadelphia, PA 19111, USA

## Abstract

**Background:**

Cadmium has been classified as a human carcinogen, affecting health through occupational and environmental exposure. Cadmium has a long biological half-life (>25 years), due to the flat kinetics of its excretion. The prostate is one of the organs with highest levels of cadmium accumulation. Importantly, patients with prostate cancer appear to have higher levels of cadmium both in the circulation and in prostatic tissues.

**Results:**

In the current report, we demonstrate for the first time that cadmium down-regulates expression of the X-linked inhibitor of apoptosis protein (XIAP) in prostate cancer cells. Cadmium-mediated XIAP depletion occurs at the post-transcriptional level via an NF-κB-independent, proteasome-mediated mechanism and coincides with an increased sensitivity of prostate cancer cells to TNF-α-mediated apoptosis. Prolonged treatment with cadmium results in selection of prostate cancer cells with apoptosis-resistant phenotype. Development of apoptosis-resistance coincides with restoration of XIAP expression in cadmium-selected PC-3 cells.

**Conclusions:**

Selection of cadmium-resistant cells could represent an adaptive survival mechanism that may contribute to progression of prostatic malignancies.

## Background

Cadmium is a ubiquitous environmental pollutant that is classified as a human carcinogen by the International Agency for Research on Cancer and the National Toxicology Program. Exposure to cadmium and cadmium-containing compounds primarily occurs in the workplace (e. g. mining, smelting, processing, product formulations, and battery manufacturing). Meanwhile, non-occupational exposure is also widespread and stems from foods and tobacco smoke [1]. Furthermore, cadmium has been detected in significant amounts in all tested zinc-containing dietary supplements [2]. Cadmium has a long biological half-life (>25 years), due to the flat kinetics of its excretion [3]. The prostate is one of the organs with highest levels of cadmium accumulation [4,5]. The carcinogenic properties of cadmium have been extensively studied, using *in vitro *cell culture and *in vivo *animal models. *In vitro *studies have reported malignant transformation of non-tumorigenic human prostate epithelial cells following cadmium exposure. The cells transformed by cadmium demonstrate morphological alterations, anchorage-independent growth in soft agar, and formation of tumors when transplanted into SCID mice [6]. In addition, cadmium chloride has been shown to produce premalignant and/or invasive epithelial lesions in the rat ventral prostate when administered in drinking water [7-9]. Interestingly, patients with prostate cancer appear to have higher levels of cadmium both in the circulation and in prostatic tissues [10]. Aberrant gene expression resulting in increased cell proliferation or blockade of apoptosis may be the mechanisms responsible for cadmium-mediated carcinogenesis [11].

Inhibitor of apoptosis proteins (IAPs) are a family of caspase inhibitors that selectively regulate the activity of both initiator and effector caspases [12,13]. In addition to the regulation of apoptosis, IAPs are also involved in various cellular functions, including cell cycle modulation, intracellular signal transduction and targeting of proteins to the ubiquitin-proteasome degradation machinery [14-16]. Of all the members of the IAP family, the X-linked inhibitor of apoptosis protein (XIAP) has received the most interest. XIAP is a 57 kDa protein with three zinc-binding baculovirus IAP repeat (BIR) domains. These domains are essential for the inhibitory activity of XIAP in apoptosis. An additional zinc-binding motif, the really interesting new gene (RING) domain, contains E3 ubiquitin ligase activity [16]. Importantly, XIAP is the only member of the IAP family that is able to directly inhibit both the initiation and execution phases of the caspase cascade [17]. Many studies have revealed a strong association between XIAP expression levels and carcinogenesis [13,17]. Elevated XIAP protein expression is described in a number of human cancers, including lymphoma [18], colon [19], lung [20], renal [21], hepatocellular [22], and prostate cells [23,24]. Increased XIAP levels have been linked to mechanisms by which cells escape anoikis and apoptosis that are induced by radiation, chemotherapy, and death receptors activation [17,23,25].

Here, we demonstrate that cadmium down-regulates the expression of XIAP at the post-transcriptional level in prostate cancer cells. The observed modulation of XIAP expression occurs via an NF-κB-independent mechanism and is due to cadmium-mediated inhibition of proteasome activity.

## Results

### Cadmium down-regulates XIAP expression at post-transcriptional level in prostate cancer cells

The ability of cadmium to substitute zinc in zinc finger domains and impair function of the wild-type zinc finger proteins has been established [26]. Given that XIAP contains three zinc-binding BIR domains and a zinc-binding RING domain, we examined the impact of cadmium on the expression of XIAP in prostate cancer cell lines.

**Figure 1:**
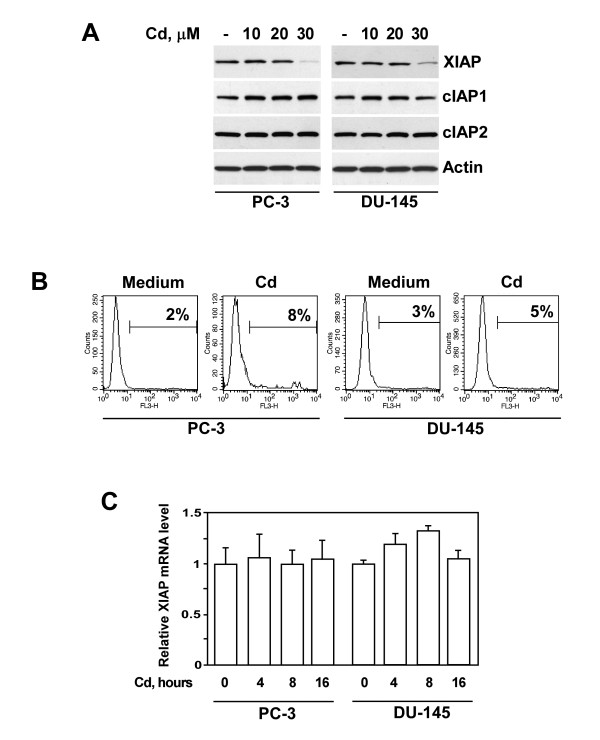
**Effect of cadmium on XIAP protein and mRNA levels in prostate cancer cells**. (A) PC-3 and DU-145 cells were treated with indicated concentrations of cadmium for 16 hours. Levels of XIAP, cIAP1 and c IAP2 proteins were detected in cell lysates by immunoblotting using specific antibodies. Expression of β-actin was used to control equal protein loading. Representative data from one of four experiments is shown. (B) The effect of cadmium on the viability of PC-3 and DU-145 cells. Cells were cultured in the absence or presence of cadmium (30 μM) for 16 hours, harvested and stained with propidium iodide followed by flow cytometry analysis. X axis represents fluorescence intensity, Y axis represents cell number. Representative data from one of three experiments. (C) PC-3 and DU-145 cells were treated with cadmium (30 μM) for indicated periods of time. XIAP mRNA levels were examined by real-time PCR analysis as described in Materials and Methods.

To establish relevance of our *in vitro *investigations, it was essential to consider whether the concentrations of cadmium used in our experiments were compatible with those that were found in target cells of exposed organisms. The concentrations of cadmium used in our study (10-30 μM) were indeed within the range documented for prostatic tissues in humans [27]. As demonstrated in Figure 1A, treatment of human PC-3 and DU-145 cells with cadmium resulted in a decrease of XIAP protein level. Inhibition of XIAP expression is selective, as cadmium had no effect on the levels of other members of IAP family, namely cIAP1 and cIAP2 (Fig. 1A). Furthermore, it is important to note that XIAP down-regulation was observed at cadmium concentrations that had no significant effect on cell viability (Fig 1B). To test the possibility that cadmium modulates XIAP expression at the transcriptional level, we examined the levels of XIAP mRNA in PC-3 and DU-145 cells incubated with cadmium by real-time PCR. As shown in Figure 1C, XIAP mRNA levels were not suppressed by cadmium.

### Modulation of XIAP expression by cadmium occurs via NF-κB-independent, proteasome-mediated mechanism

**Figure 2:**
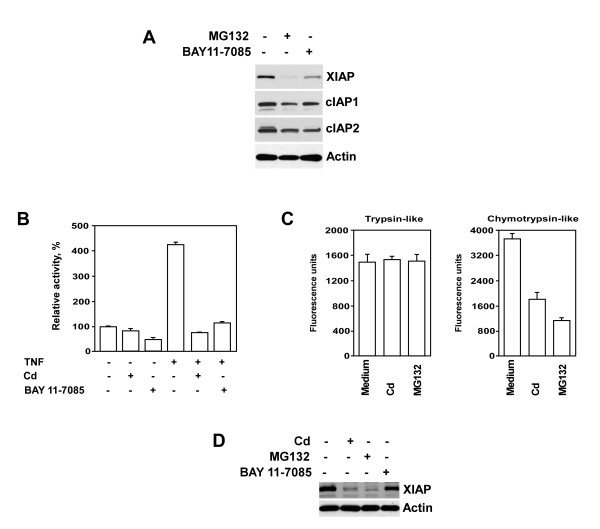
**Modulation of XIAP expression by cadmium occurs via NF-kappaB–independent, proteasome-mediated mechanism. **(A) The effect of NF-κB inhibitor BAY 11-7085 and proteasome inhibitor MG132 on the expression of XIAP, cIAP1 and cIAP2 in PC-3 prostate cancer cells. Cells were treated with either BAY 11-7085 (5 μM) or MG132 (5 μM) for 16 hours. Levels of XIAP, cIAP1 and c IAP2 proteins were detected in cell lysates by immunoblotting using specific antibodies. Expression of α-actin was used to control equal protein loading. (B) Luciferase reporter assay of NF-κB activity in PC-3 cells. Cells were pre-incubated with cadmium (30 μM) or NF-κB inhibitor BAY 11-7085 (5 μM) for 3 hours followed by incubation with or w/o TNF-α (20 ng/ml) for an additional 4 hours. Columns, means of three different samples; bars, SEM. (C) Analysis of proteasome activity in PC-3 cells. Cells were incubated with cadmium (30 μM) or the proteasome inhibitor MG132 (5 μM) for 3 hours. Chymotrypsin- and trypsin-like activities were examined as described in Materials and Methods. Columns, means of three different samples; bars, SEM. (D) PC-3 cells were transfected with the N-terminally HA-tagged XIAP construct under the control of the NF-κB independent SV40 promoter. Four hours after transfection cell culture medium was replaced with medium containing either cadmium (30 μM) or MG132 (5 μM) or BAY 11-7085 (5 μM) and cells were cultured for additional 16 hours. Expression of XIAP and α-actin was detected by immunoblotting with anti-HA or anti-actin antibodies respectively. Representative data from one of three experiments is shown.

XIAP is a protein that is regulated by NF-κB [28]. Involvement of the proteasome pathway in post-translational regulation of IAPs likewise has been demonstrated [18,29,30]. Data from our laboratory, shown in Figure 2A, demonstrate that both the NF-κB inhibitor BAY 11-7085 and the proteasome inhibitor MG132 markedly reduce XIAP expression in PC-3 prostate cancer cells. Importantly, reduction of XIAP expression was not secondary to a rise in cell death. The viability of cells following co-culture with either BAY 11-7085 or MG132 exceeded 85%, as examined by propidium iodide staining followed by flow cytometry analysis (data not shown). Notably, expression levels of cIAP1 and cIAP2 proteins were also slightly reduced in these cells. Inhibitory effects of BAY 11-7085 and MG132 on the NF-κB transcriptional activity may be responsible for this finding. Recent studies document discrepant data on the effect of cadmium on the activation of NF-κB. Some reports demonstrate cadmium-induced inhibition of NF-κB binding to DNA [31], while others note an increase in NF-κB activity in cells treated with cadmium [32,33]. Therefore, we examined the effect of cadmium on the status of NF-κB activity in PC-3 prostate cancer cells using the luciferase reporter assay. The findings presented in Figure 2B demonstrate that NF-κB activation was markedly reduced in cells pre-treated with either cadmium or NF-κB inhibitor BAY 11-7085. Nevertheless, given that XIAP mRNA levels were not suppressed in cadmium-treated cells (Fig. 1C), the results of our experiments suggest that NF-κB-controlled pathway is not involved in cadmium-mediated down-regulation of XIAP protein levels in prostate cancer cells. Importantly, cadmium either alone (Fig. 1B) or in combination with TNF-α (data not shown) did not affect cell viability at the time point studied.

To examine the possibility that cadmium modulates XIAP expression via proteasome-dependent mechanisms, we first examined trypsin- and chymotrypsin-like proteasome activities in PC-3 cells cultured in the presence of cadmium. As demonstrated in Figure 2C, cadmium significantly reduced only chymotrypsin-like proteasome activity in PC-3 cells. Similar results were obtained with a proteasome inhibitor MG132 (Fig. 2C). MG132 is a peptide aldehyde that acts as a potent competitive inhibitor of the chymotrypsin-like activity of the proteasome complex [34]. To validate further the possibility that cadmium down-regulates the expression of XIAP at the post-transcriptional level via proteasome-mediated NF-κB-independent mechanism, we transfected PC-3 cells with the N-terminally HA-tagged XIAP constructs under the control of the NF-κB-independent SV40 promoter [35]. As demonstrated in Figure 2A and 2D, cadmium completely blocked XIAP expression irrespective of the transcriptional origin. This was documented by Western blotting analysis using the anti-HA antibody. In addition, pre-treatment with a proteasome inhibitor MG132 also completely blocked expression of XIAP, whereas treatment with NF-κB inhibitor, BAY 11-7085, had no effect on the XIAP expression. Therefore, the results of our experiments reveal that cadmium down-regulates expression of XIAP through an NF-κB-independent, proteasome-mediated mechanism.

**Figure 3:**
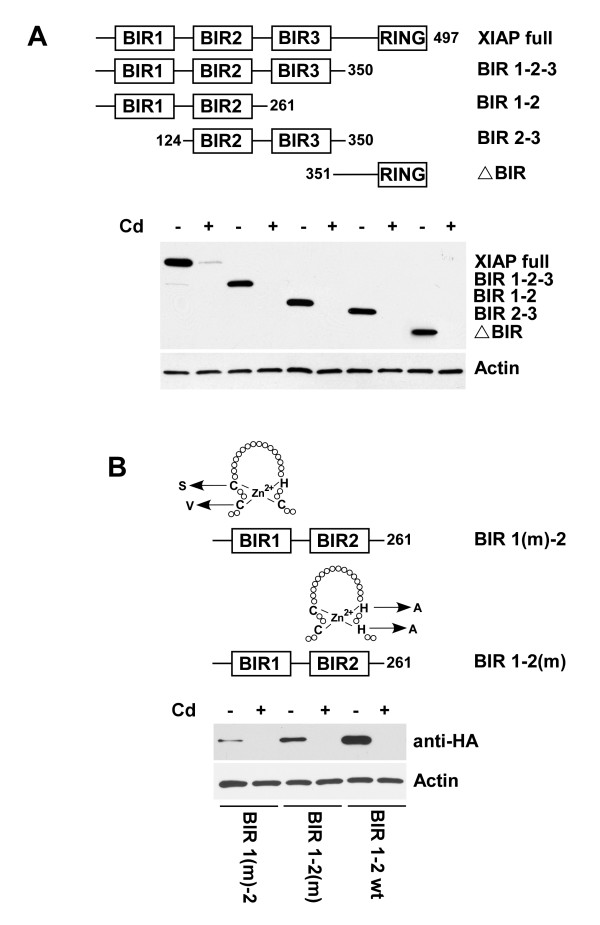
**Cadmium blocks expression of various XIAP domains**. (A) PC-3 cells were transfected with the indicated HA-tagged XIAP constructs or (B) BIR 1-2 constructs with point mutations in zinc binding sites under the control of the SV40 promoter. Four hours after transfection cell culture medium was replaced with medium containing cadmium (30 μM) and cells were cultures for an additional 16 hours. Expression of XIAP truncated mutants and α-actin was detected by immunoblotting with anti-HA or anti-actin antibodies, respectively. Representative data from one of three experiments.

The potential sensitivity of various domains of XIAP to cadmium-mediated down-regulation was also examined in PC-3 cells. These cells were transfected with the appropriately truncated constructs containing N-terminal HA-tag under the control of SV40 promoter. As shown in Figure 3A, the expression of all examined XIAP domains was reduced in the presence of cadmium, as was the expression of the full length XIAP protein. Similar results were obtained with XIAP constructs carrying mutations of the zinc binding sites. As demonstrated in Figure 3B, mutations of zinc binding sites didn't protect XIAP from cadmium-mediated depletion.

**Figure 4:**
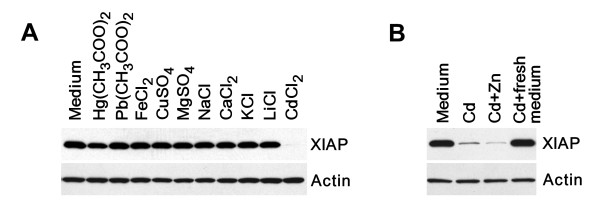
**The effect of metal ions on XIAP expression. **(A) Expression of XIAP in prostate cancer cells was prevented by cadmium only but not by other tested metal ions. PC-3 cells were transfected with the HA-tagged XIAP construct under the control of the SV40 promoter. Four hours after transfection cell culture medium was replaced with medium containing 30 μM of indicated metal salts and cells were cultured for additional 16 hours. Expression of XIAP and α-actin was detected by immunoblotting with anti-HA or anti-actin antibodies respectively. (B) PC-3 cells were transfected with the HA-tagged XIAP construct under the control of the SV40 promoter. Four hours after transfection cell culture medium was replaced with medium containing cadmium (30 μM) and cells were cultured for an additional 16 hours with or without equimolar concentration of zinc. In an additional set of experiments, cells were first incubated in cadmium-containing medium. After 16 hours, all medium containing cadmium was removed and replaced with fresh medium. Cells were then incubated for an additional 4 hours in the absence of cadmium.

Given that other D-block transition metals such as copper can also modulate XIAP expression at the post-transcriptional level [36], cell culture medium was supplemented with various metal ions. As demonstrated in Figure 4A, the addition of cadmium, but not other metals was responsible for reduction of XIAP expression. Cadmium belongs to the same chemical group as the metal zinc and competes with this element for cysteinyl clusters in many proteins [26,37]. Therefore, we examined whether the supplementation with zinc could restore XIAP expression in the presence of cadmium. The results presented in Figure 4B demonstrate that the addition of zinc failed to restore XIAP expression in cadmium-treated cells. Furthermore, to determine whether down-regulation of XIAP expression is a reversible process, we replaced cadmium-containing medium with fresh medium without cadmium. Depletion of cadmium from the cell culture medium completely restored XIAP expression in PC-3 cells after 4 hours (Fig. 4B).

### Cadmium sensitizes prostate cancer cells to TNF-α-mediated apoptosis

**Figure 5:**
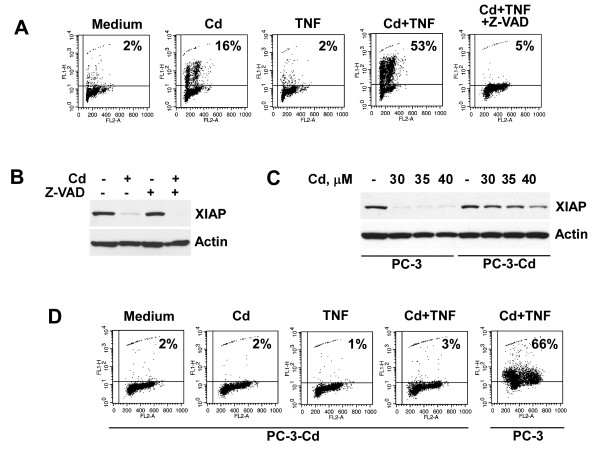
**Cadmium sensitizes prostate cancer cells to TNF-alpha-mediated apoptosis.** (A) PC-3 cells were pre-treated with cadmium (30 μM) for 16 hours followed by stimulation with TNF-α (20 ng/ml) with or without Z-VAD.FMK (50 μM) for an additional 20 hours. The percentage of apoptotic cells was determined by the APO-BRDU assay followed by flow cytometry analysis. Representative data from one of three experiments is shown. (B) PC-3 cells were pre-incubated with or without Z-VAD.FMK (50 μM) for 30 minutes followed by treatment with cadmium (30 μM) for 16 hours. XIAP expression was detected in cell lysates by immunoblotting with specific antibody. (C) PC-3 cells were cultured in medium alone or in the presence of cadmium (30 μM) for 7 days followed by treatment with indicated concentrations of cadmium for 16 hours. XIAP expression was detected in cell lysates by immunoblotting. (D) Aliquots of cells described in panel C were pre-treated with cadmium (30 μM) for 16 hours followed by stimulation with TNF-α (20 ng/ml) for an additional 20 hours. The percentage of apoptotic cells was determined by the APO-BRDU assay. Representative data from one of three experiments is shown.

The inhibition or down-regulation of XIAP lowers the apoptotic threshold [17,38,39]. Therefore, we evaluated the pro-apoptotic effect of cadmium in combination with TNF-α on prostate cancer cells. Treatment with TNF-α alone had no significant effect on cell death in PC-3 cells, as prostate cancer cells are generally resistant to TNF-α-mediated apoptosis. Treatment with cadmium alone induced apoptosis only in 16% of cells as was detected by the TUNEL assay. Concomitant treatment with TNF-α and cadmium, on the other hand, induced apoptosis in 53% of cells (Fig. 5A). We demonstrated previously that direct small interfering RNA-mediated knockdown of XIAP results in sensitization of PC-3 cells to death ligand-mediated apoptosis, suggesting that depletion of XIAP by itself is sufficient to reverse apoptosis resistance in prostate cancer cells [40]. Recent studies reveal that caspase family members induce XIAP cleavage under stress conditions [41]. The inhibition of caspase activity with the pan-caspase inhibitor Z-VAD-FMK efficiently blocked apoptosis in PC-3 cells concomitantly treated with TNF-α and cadmium (Fig. 5A). However, Z-VAD-FMK did not block the ability of cadmium to reduce the XIAP protein levels (Fig. 5B), indicating that cadmium-mediated down-regulation of XIAP protein levels is a caspase-independent process.

Acquisition of apoptosis-resistance can potentially contribute to progression of prostatic malignancies. We investigated whether prolonged treatment with cadmium results in selection of prostate cancer cells with cadmium-resistant phenotype. PC-3 cells were pre-treated with cadmium at 30 μM for 7 days. As demonstrated in Figure 5C, expression of XIAP in cadmium-selected PC-3 cells (PC-3-Cd) was not modified after treatment with escalating concentrations of cadmium, whereas cadmium completely reduced XIAP protein levels in parental PC-3 cells at all tested concentrations. Moreover, cadmium-selected PC-3 cells were also resistant to apoptosis in response to concomitant treatment with TNF-α and cadmium (Fig. 5D). Our experiments demonstrate that indeed cadmium treatment results in the establishment of cadmium resistant phenotype in prostate cancer cells.

## Discussion

Cadmium is a toxic heavy metal. Its wide use in industry is suspected to have widespread deleterious effects on human health through occupational and environmental exposure. The element contributes to pathogenesis of osteoporosis, non-hypertrophic emphysema, irreversible renal tubular injury, anemia, eosinophilia, anosmia and chronic rhinitis. In addition, cadmium is a potent human carcinogen and occupational exposure to the metal is associated with cancers of the lung, prostate, pancreas, and kidney [42]. The biological half-life of cadmium in humans is estimated to be >25 years, thereby assuring the metal's accumulation in tissues during one's lifetime [3]. Therefore, cadmium progressively accumulates in the human prostate with increasing age. Indeed, the prostate is an organ with one of the highest levels of cadmium [4,5].

Multiple mechanisms of carcinogenesis for cadmium have been suggested. These include aberrant gene expression, inhibition of DNA damage repair, induction of oxidative stress, and inhibition of apoptosis [43]. In contrast, some studies demonstrate that cadmium can inhibit the formation of chemically induced and spontaneously occurring tumors in animals when given at non-toxic concentrations [44,45].

The present study provides the first evidence that cadmium induces depletion of XIAP in prostate caner cells at the post-transcriptional level via proteasome-mediated mechanisms. The inhibition or down-regulation of XIAP in cancer cells lowers the apoptotic threshold, thereby inducing cell death and/or enhancing the cytotoxic action of chemotherapeutic agents. Recent studies demonstrate that XIAP antagonist 1396-34 sensitizes PC-3 and DU-145 prostate cancer cells to chemotherapeutic agents and TRAIL [46]. In accord with these reports, our data show that cadmium-mediated down-regulation of XIAP coincides with increased sensitivity of PC-3 prostate cancer cells to TNF-α-mediated apoptosis.

Cadmium induces generation of reactive oxygen species in target cells with subsequent mitochondrial damage [47]. Mitochondrial collapse results in the release of various apoptogenic factors into the cytoplasm. These include IAP-binding proteins Smac/DIABLO and Omi/HtrA2 [48]. Interestingly, both Smac/DIABLO and Omi/HtrA2 are capable of inducing caspase-independent degradation of IAPs including XIAP, cIAP1 and cIAP2 [49-51]. However, the potential involvement of Smac/DIABLO and Omi/HtrA2 in cadmium-mediated XIAP depletion could be excluded based on the findings that Smac/DIABLO selectively reduces the protein levels of cIAP1 and cIAP2 but not that of XIAP [51], whereas Omi/HtrA2 induces proteolytic cleavage of all IAPs (i.e. XIAP, cIAP1 and cIAP2) [50]. Importantly, cadmium-mediated depletion of XIAP was selective, as cadmium had no effect on the levels of other members of the IAP family, namely cIAP1 and cIAP2 (Fig. 1A).

Selection of apoptosis-resistant cells is a potential mechanism for tumor progression. Results of our experiments reveal that pre-treatment with cadmium produces development of apoptosis-resistance in response to concomitant treatment with TNF-α and cadmium in prostate cancer cells (Fig. 5D). Development of apoptosis-resistance coincides with restoration of XIAP expression in cadmium-selected PC-3 cells. Interestingly, while some studies demonstrate that high levels of XIAP have an unfavorable prognosis in cancers of various tissue origins [21,52], other data suggest that elevated XIAP levels are associated with a favorable clinical outcome [53,54]. Likely, XIAP expression alone cannot serve as a predictive marker of chemoresistance. Given that tumorigenesis is a complex multifactorial process, expression levels and functional states of other critical pro- and anti-apoptotic molecules must be integrated for accurate prognostication. Recent studies by Seeger et al. demonstrate that the finely tuned balance between XIAP and its antagonists is critical in determining the clinical outcome in cancer patients [55].

A potential explanation for the development of cadmium-resistant phenotype in prostate cancer cells is increased expression of metallothioneins in response to cadmium treatment, resulting in the intracellular chelation of cadmium ions. Albrecht et al. demonstrated that exposure of the normal prostate cells to cadmium results in the rapid induction of the various metallothionein isoforms with eventual accumulation easily exceeding 10% of total cellular protein. Moreover, maximum accumulation of metallothioneins was detected on days 7-13 after the start of treatment [56].

Recent reports suggest that several metals may modulate XIAP integrity. For instance, elevated copper levels result in a conformational change in XIAP, which accelerates its degradation. Importantly, copper does not reduce XIAP mRNA expression [36]. These data indicate that both cadmium and copper modulate XIAP expression at the post-transcriptional level. Despite these published reports in cell culture of other tissues, our experiments suggest that copper's effects on XIAP expression in prostate tissues are not as significant, since pre-treatment with copper had no effect on XIAP expression in prostate cancer cells (Fig. 4A).

Given cadmium's chemical similarity to zinc, a possibility exists that cadmium exchanges for zinc and leads to instability of the XIAP protein. The selective antagonism by zinc of the carcinogenic effect of cadmium suggests that zinc may act at a variety of important binding sites, including those that are potentially important for regulation of gene expression or of the enzyme's catalytic activity. Indeed, the ability of cadmium, to substitute zinc in the zinc finger domains and impair function of the wild-type zinc finger proteins has been demonstrated [26]. Cadmium's substitution for zinc in the tumor suppressor protein, p53 alters p53 conformation and results in loss of DNA binding capacity and suppression of p53-dependent cell cycle arrest. Zinc supplementation, on the other hand, reactivates p53 and restores its tumor suppressive functions [26]. Indeed, zinc and cadmium are the only two metal ions that appear to effect cellular XIAP levels. In contrast to cadmium, it is the depletion of zinc that leads to cellular reduction of XIAP [40]. Nevertheless, in contrast to data available for p53, zinc supplementation in our hands failed to restore expression of XIAP in cells treated with cadmium (Fig. 4B).

XIAP is one of the NF-κB-regulated proteins [28]. Nevertheless, our data suggest that suppression of XIAP expression by cadmium is likely an NF-κB-independent process. Our work shows that cadmium reduces XIAP levels even when the protein is expressed under an NF-κB-independent SV40 promoter. Instead, the mechanism for XIAP suppression may be proteasome-dependent.

## Conclusions

In this study we investigated the molecular mechanisms of cadmium-mediated XIAP down-regulation in prostate cancer cells. Our results demonstrate that cadmium down-regulates the expression of XIAP at post-transcriptional level via an NF-κB-independent mechanism. Furthermore, our work reveals the critical role of a proteasome-dependent mechanism for the cadmium-mediated modulation of XIAP expression.

## Methods

### Cells and materials

Cell lines were obtained from ATCC (Rockville, MD) and maintained in RPMI 1640 medium (Bio-Whittaker, Walkersville, MD) supplemented with 10% FBS (Hyclone, Logan, UT), gentamicin (50 mg/L), sodium pyruvate (1 mM) and non-essential amino acids (0.1 mM).

### Antibodies and Reagents

Antibody to HA-tag was obtained from Santa Cruz Biotechnology (Santa Cruz, CA). Antibody to XIAP was obtained from Cell Signaling Technology (Beverly, MA). TNF-α and antibody to β-actin were obtained from Sigma (St. Louis, MO). MG132, BAY 11-7085 and Z-VAD.FMK were obtained from Biomol (Plymouth Meeting, PA).

### Western Blot Analysis

Cells were lysed in a boiling SDS buffer (66 mM Tris-HCl (pH 7.5), 2% SDS) for 10 minutes. SDS-PAGE and Western blotting were performed as previously described [57].

### Real-Time PCR analysis

Total RNA was isolated from cells using Mini RNA isolation II Kit (Zymo Research, Orange, CA) following with DNase I (New England Biolabs, Ipswich, MA) treatment. RNA was purified using RNA Clean and Concentrator Kit (Zymo Research). Total RNA (1 μg) was reverse transcribed in final volume of 20 μl with 100 U of Superscript III Reverse Transcriptase (Invitrogen, Gaithersburg, MD) according to the manufacturer's instructions. After reverse transcription cDNA samples were diluted 40 fold, and 5 μl of diluted cDNA were amplified by real-time PCR using the XIAP TaqMan Gene Expression Assay (ID# Hs00745222_s1). GAPDH Gene Expression Assay (ID# Hs99999905_m1) was used as endogenous control. Each sample was run in triplicate for both XIAP and GAPDH in 20 μl reaction using TaqMan Gene Expression Master Mix according to the manufacturer's instructions (Applied Biosystems, Foster City, CA). Reactions were carried out in an Applied Biosystems 7500 real-time PCR System. Analysis of relative XIAP gene expression data was carried out using the 2^-ΔΔCT ^method [58].

### Plasmids and transfection

pEBB-HA-XIAP vector and XIAP truncation mutants were a kind gift from Dr. C. S. Duckett (University of Michigan Medical School, Ann Arbor, MI). Transfections were performed using TransIT-Prostate transfection kit (Mirus Bio, Madison, WI).

### Luciferase reporter assay

Cells were transfected with pNF-κB-luc (Stratagene, La Jolla, CA) and pRL-TK (Promega, Madison, WI) plasmids. Twenty-four hours after transfection, cells were treated with cadmium (30 μM) or NF-κB inhibitor BAY 11-7085 (10 μM) for 3 hours followed by treatment with TNF-α (20 ng/ml) for an additional 4 hours. Samples were assayed for firefly and renilla luciferase activities using the Dual-Glo Luciferase assay System (Promega) and normalized as instructed by the manufacturer.

### Zinc finger mutagenesis

The strategies for nonfunctional zinc finger mutation have been described previously [59]. C63V and C66S mutations of zinc finger in BIR1 domain of XIAP BIR1-2 polypeptide were generated by PCR with overlapping internal primers spanning the mutated region and external primers containing restriction sites (underlined in primer ssequences) for the cloning into the pEBB vector. BIR1-2 ORF was used as a template. Briefly, two PCR products were amplified using first forward 5'-atcttgggatccATGACTTTTAACAGTTTTGAAGG and reverse 5'-TGtgaACTAAAgacCCGCACGGT primer pairs and second forward 5'-ACCGTGCGGgtcTTTAGTtcaCA and reverse 5'-atcttgatcgat TTAGGATGGATTTCTTGGAAGATTTG primer pairs. These PCR products were purified, diluted, mixed and re-amplified with external primers for subsequent cloning into pEBB vector. The same strategy was used to generate H220A and H223A mutations in the BIR2 domain of XIAP BIR1-2 polypeptide. Overlapping internal primers had 5'-GAAgccAGGCGAgccTTTCCTAATT and 5'-AATTAGGTTTggcTCGCCTggcTTC sequences, while external primers remained the same. All mutations were confirmed by sequencing in the Fox Chase Cancer Center Automated DNA Sequencing Facility.

### Measurement of apoptosis

DNA fragmentation was detected using APO-BRDU kit (The Phoenix Flow Systems, Inc., San Diego, CA).

### Measurement of proteasome activity

Cells were lysed in a buffer (50 mM Tris (pH 7.6), 150 mM NaCl, 1% Triton X-100). Protein concentration was measured with a commercial kit (Bio-Rad, Richmond, CA). The proteasome activity assay was performed in 96-well plates by incubating 10 μg of proteins with fluorescent proteasome substrates (Biomol, Plymouth Meeting, PA) as suggested by the manufacturer.

## Competing interests

The authors declare that they have no competing interests.

## Authors' contributions

KG carried out immunobloting and drafted the manuscript. PM performed real-time PCR analysis. RGU contributed to the concept and design of the study and helped to draft the manuscript. AK carried out flow cytometry analysis. DJK and EF examined proteasome activity and performed luciferase reporter assay. VMK conceived the studies, oversaw the experimental work and finalized the manuscript. All authors have read and approved the final manuscript.
